# Long-Term Effects of Gestational Nicotine Exposure and Food-Restriction on Gene Expression in the Striatum of Adolescent Rats

**DOI:** 10.1371/journal.pone.0088896

**Published:** 2014-02-19

**Authors:** Nicholas E. Ilott, Tomasz Schneider, Jonathan Mill, Leonard Schalkwyk, Giovana Brolese, Lisiane Bizarro, Ian P. Stolerman, Emma Dempster, Philip Asherson

**Affiliations:** 1 Medical Research Council (MRC), Social, Genetic and Developmental Psychiatry (SGDP) centre, Institute of Psychiatry, King's College London, London, United Kingdom; 2 Computational Genomics Analysis and Training (CGAT), Medical Research Council (MRC) Functional Genomics Unit, Department of Physiology, Anatomy and Genetics, University of Oxford, Oxford, United Kingdom; 3 Department of addictions, Institute of Psychiatry, King's College London, London, United Kingdom,; 4 Experimental Psychology, University of Oxford, Oxford, United Kingdom; 5 University of Exeter Medical School, Exeter, United Kingdom; 6 Departamento de Psicologia do Desenvolvimento e da Personalidade, Instituto de Psicologia, Universidade Federal do Rio Grande do Sul, Porto Alegre-RS, Brazil; Universidade do Estado do Rio de Janeiro, Brazil

## Abstract

Gestational exposure to environmental toxins such as nicotine may result in detectable gene expression changes in later life. To investigate the direct toxic effects of prenatal nicotine exposure on later brain development, we have used transcriptomic analysis of striatal samples to identify gene expression differences between adolescent Lister Hooded rats exposed to nicotine *in utero* and controls. Using an additional group of animals matched for the reduced food intake experienced in the nicotine group, we were also able to assess the impact of imposed food-restriction on gene expression profiles. We found little evidence for a role of gestational nicotine exposure on altered gene expression in the striatum of adolescent offspring at a significance level of p<0.01 and |log2 fold change >0.5|, although we cannot exclude the possibility of nicotine-induced changes in other brain regions, or at other time points. We did, however, find marked gene expression differences in response to imposed food-restriction. Food-restriction resulted in significant group differences for a number of immediate early genes (IEGs) including *Fos, Fosb, Fosl2, Arc, Junb, Nr4a1* and *Nr4a3*. These genes are associated with stress response pathways and therefore may reflect long-term effects of nutritional deprivation on the development of the stress system.

## Introduction

Smoking during pregnancy (SDP) is associated with a variety of neonatal perturbations including low birth weight [1]–[6] and sudden infant death syndrome (SIDS) [7], as well as later-developing behavioural outcomes [4], [8]–[10]. Animal studies have identified both physical and behavioural abnormalities due to prenatal nicotine exposure, including delayed development and maturation [11]–[13], hyperactivity [14], [15], increased markers of anxiety [16], as well as cognitive performance deficits that reflect aspects of impulse control and attention regulation in both adult and adolescent animals [17], [18]. The molecular basis of these associations may involve alterations of the cholinergic system, where the stimulation of nicotinic acetylcholine receptor (nAChR) subunits is important in a variety of neuronal processes throughout development [19]. Changes in gene expression of nAChR subunits and regulators of acetylcholine neurotransmission have been observed in animals prenatally exposed to nicotine [20]–[22]. Links between nAChR function and the dopamine system are also highlighted by the observation of an increase in dopamine receptor D5 *(Drd5)* mRNA expression in the striatum of adult rats gestationally exposed to nicotine [18], as well as a reduced nicotine-induced dopamine release in adolescent rats [23]. Further, the first large-scale gene expression studies using custom, pathway focused microarrays have revealed a role for cell adhesion and cell death systems in limbic brain regions of adolescent rats exposed to nicotine *in utero* [24], [25].

A potential problem with studying the effects of gestational nicotine on molecular systems is that nicotine administration additionally affects food intake, weight gain and fluid intake [13], [18], [26], [27], resulting in a reduced ability to distinguish the effects of nicotine from those of nutrition or other confounders. Matching food intake to nicotine-exposed animals via experimental controls may alleviate these problems. However, both undernutrition and the likely stress that is introduced by imposed food-restriction may also affect gene expression profiles via their effect on hypothalamic-pituitary-adrenal (HPA) axis development [28]. While gestational food-restriction and nicotine exposure are associated with some behavioural similarities (i.e. increased nicotine consumption and omission errors in the 5-choice serial reaction time task [5-CSRTT]), there exist substantial behavioural differences between these groups [17]. For example, nicotine exposed offspring display hyperactivity, increased number of open arm entries in the elevated plus maze and increased numbers of anticipatory responses in the 5-CSRTT. This suggests that while there may be shared molecular signatures, a significant proportion is likely to be distinct. Nevertheless, any differences due to gestational food-restriction remain of considerable interest. It has been established that growth restriction induced by maternal undernutrition has long-term effects on mRNA expression in offspring. For example, sirtuin 1 (*Sirt1*) is reduced [29] and somatic cytochrome c (*CYCS*) [30] is increased in the liver of offspring gestationally exposed to energy restriction. Further, hypothalamic regulation of metabolic processes may also be altered, as evidence suggests increased expression of the leptin receptor and fat mass and obesity-associated (*FTO*) gene in gestationally malnourished offspring hypothalamus [31], [32]. The effects of maternal undernutrition on gene expression in the striatum, however, have not yet been studied.

To further our understanding of the long-term effects of gestational nicotine exposure on brain development, we have conducted the first genome-scale mRNA expression profiling study in adolescent rats exposed to nicotine *in utero*. The analyses have been performed in the striatum, a brain structure that is sensitive to nicotine exposure [33] and is implicated in the development of ADHD [34]–[38].

To control for potential confounding effects of differences in food intake between the nicotine-exposed group and controls, we included an additional group of animals that were matched for food-intake with the nicotine-exposed group. Given the current literature, we hypothesised that the nicotine group would show dysregulation of genes involved in nAChR receptor signaling and dopamine function. In addition, we expected any differences observed due to food-restriction would be distinct from the effects of nicotine [33] yet may reflect a disturbance in the development of metabolic or stress related pathways.

## Materials and Methods

### Animals

The treatment of animals complied with the UK Animals (Scientific Procedures) Act 1986 and the Code of Practice of the Institute of Psychiatry. The work was carried out under the Project License PPL 70/5569 “Psychopharmacology of nicotine dependence” issued by the Home Office of the British Government in 2003 and amended in 2006. Nicotine was administered in the drinking water of pregnant rats in order to avoid stress of invasive surgical procedures involving implants. Supplementary water was made available for drinking to minimise the consequent reductions in fluid intake. The minimum number of animals consistent with adequate statistical power was used. Animals were sacrificed by cervical dislocation.

Both male and female Lister hooded rats (Harlan Olac, Bicester, UK) were used. They were housed individually (except during mating) in a temperature (21±2^°C^) and humidity (50±10%) controlled environment on a 12 h light–dark cycle (lights on from 0700 h) and had *ad libitum* access to drinking fluids (tap water or nicotine solutions).

Females were divided into three separate groups according to a randomised block design balanced to their body weight into: nicotine exposure (Nic, n = 8), control group (Con, n = 8) and a food-restricted (pair-fed) group (Con-Pf, n = 9) that was matched for food intake to the Nic animals, was also established. Pair-fed animals were provided each day with the amount of food eaten by matched nicotine-exposed animals on the previous day; their access to water was not restricted.

Nicotine bitartrate (Sigma, USA) was dissolved in the drinking water at varying concentrations and nicotine-containing water was adjusted to the pH of drinking water with 0.001 NaOH. Habituation to increasing concentrations of nicotine solution (0.02, 0.04, 0.06 mg/ml) as the only source of fluid was introduced to the Nic exposed group 3-weeks before mating.

Females were controlled according to their oestrous cycle. Females in pro-oestrus and oestrous were mated during the dark phase of the day at the beginning of the fourth week of nicotine exposure. Nicotine solution was not withheld before mating. The day on which a vaginal plug or spermatozoa in the vaginal smear were found was defined as gestational day 0.

Pregnant females from nicotine, control and pair-fed groups were weighed twice weekly. Consumption of nicotine solution was assessed on a daily basis. Females drinking less than 10 ml of nicotine solution on any particular day were given access to tap water for 3 min. Food consumption was evaluated 3 times a week.

All dams were checked twice daily (before 8am and after 4.30pm) starting a few days before delivery. Deliveries completed by 8am were assigned to postnatal day 1 (PND1). Pups born later that day were assigned to PND1 on the following morning. Litters were examined on PND1 for obvious morphological anomalies (e.g., missing digits, facial malformations, etc.), sexed by relative ano-genital distance and, in the case of litters with more than 8 offspring, culled randomly to 8 pups with equal numbers of males and females per litter whenever possible. 8–9 litters were used to assess birthweight of offspring from each group. The dam was first removed from the home cage and birthweight measurements were taken between 9.00am and 4.00pm.

Our previously published work provides additional details regarding the nicotine administration protocol used in the present study [17]. Briefly, using this protocol we obtained 81.1±23.2 ng/ml blood nicotine in those dams assessed. There were no significant effects of nicotine on litter characteristics: number of animals per litter, the numbers of females and males per litter and sex ratio (see [17] for details).

Data concerning maternal fluid intake, food consumption and offspring bodyweight were analysed for between group differences using 1-way ANOVA. Significant differences between the three groups were further assessed *post-hoc* using Tukey's Honest Significant Difference test (HSD), implemented in R2.10.1.

### Sample Preparation

Experimentally naïve male offspring, littermates of animals used in the previously published behavioural study [17] from the three gestational conditions were used for microarray analysis: a) prenatal nicotine exposed (Nic, n = 8), b) controls (Con, n = 10) and c) pair-fed (Con-Pf, n = 10) resulting in a total of n = 28. Results presented here (e.g. in Table 1) are for this subset of the larger number of offspring generated for the previously published behavioural study; the data in Table 1 are therefore derived from, but not the same as, those in [17].

**Table 1:** Nicotine administration and food-restriction have effects on pregnancy and birth characteristics.

Measure	Timing	F	P-value	Con	Nic	Con-Pf
Dams						
Mean body weight	Week before pregnancy	12.38	2.50E-04	252.2±6.2	213.9±6.5*	223.4±4.1*
(g)	Pregnancy	13.30	1.63E-04	289.1±6.7	247.8±6.8*	260.3±3.5*
Mean food consumption	Week before pregnancy	13.06	1.83E-04	15.6±0.4	13.3±0.4*	13.7±0.0*
(g/day)	Pregnancy	10.23	7.24E-04	21.4±0.4	17.8±0.9*	19.3±0.0*
Mean food consumption	Week before pregnancy	0.05	0.95	61.9±1.3	62.3±1.5	61.7±1.2
(g/Kg bodyweight/day)	Pregnancy	0.82	0.46	74.3±1.4	71.7±2.3	74.2±1.1
Mean sol^n^ consumption	Week before pregnancy	19.14	1.53E-05	24.9±1.2	14.5±0.7*#	18.9±1.4*
(ml)	Pregnancy	33.40	2.16E-07	45.0±2.7	21.8±1.7*#	38.0±1.6
Mean sol^n^ consumption	Week before pregnancy	10.97	4.97E-04	98.5±3.6	68.2±3.4*#	84.5±5.7
(ml/Kg bodyweight/day)	Pregnancy	33.56	2.07E-07	155.1±6.7	88.1±6.2*#	146.0±5.8
Offspring						
Bodyweight (g)	PND1	5.8	9.08E-03	5.3±0.1	4.7±0.1*	5.1±0.1

*p<0.05 compared to Con, #p<0.05 compared to Con-Pf.

Rats were killed at post-natal day (PND) 35 to 42 by decapitation and brains were immediately dissected. Equal numbers of animals from each group were extracted on the same day. The striatum was removed, snap frozen on dry ice and stored at −80°C until RNA extraction. RNA was extracted using Qiagen AllPrep RNA/DNA minikits (Qiagen, UK) and treated with an RNase-free DNase1 to eliminate genomic DNA contamination. Purity and quality of total RNA samples was assessed using the NanoDrop Spectrophotometer and Agilent RNA 6000 pico kit (Agilent, UK), according to the manufacturer's instructions (see File S1).

### Microarray Processing

cDNA conversion and microarray hybridisation was performed using standard protocols provided by the manufacturers (File S1). To avoid possible batch effects, samples were spread across hybridisation date, fluidics machine and fluidics module in a balanced manner (details available from corresponding author). Microarrays used in the current study were the Affymetrix rat GeneChip 1.0st array. 2 Con samples were removed after array scanning due to clear data quality issues. An outlying sample (from Nic group) was additionally removed after data processing.

### Statistical analysis of microarray data

The Robust Multichip Algorithm (RMA) [39] as implemented in the affy package from Bioconductor was used to quantile normalise the expression data and normalised expression summaries were used for all downstream analyses conducted in the statistical software package R version 2.10.1. 25 arrays were included for downstream analysis (Nic = 7, Con = 10, Con-Pf = 8). Using the *genefilter* package in the Bioconductor suite, all probe-sets with intensity less than the median for the 25 arrays were removed from the data to ensure that non-expressed genes were not being analysed. This filtering procedure left 14,073 probe-sets for further analysis. Quality control metrics were employed to assess microarray data quality (Methods S1 in File S1 and Figure S1 in File S1).

The 25 arrays displayed similar expression profiles, with Pearson Product moment correlations between each array ranging from 0.97 to 0.99. The Shapiro-Wilk test of normality on the data rejected the normal distribution for 25% of probes at the p<0.05 level. Given that the majority of probe-sets were normally distributed, we continued downstream analysis without transforming the data further, consistent with approaches used by other groups [40].

Pair-wise mean differences in gene expression between groups were analysed using the Student's t-test. Following recommendations from the MicroArray Quality Control (MAQC) project [41] we used a combination of fold change and p-value thresholds to define significant differential expression, defined as a |log2 fold-change of ≥0.5| in combination with a p-value of p<0.01.

### Quantitative reverse transcription PCR (qRT-PCR) analysis of differentially expressed genes

Ten genes (*Fos, Fosl2, Dusp1, Arc, Junb, Egr2, Nr4a3, Nr4a1, Slc25a5* and *Npas4*) were chosen for validation using qRT-PCR. These genes were chosen as they represented genes that were sensitive to imposed food-restriction i.e. they were up-regulated in the Con-Pf group when compared to both Con and Nic groups. Assays were performed with inventoried TaqMan assays (Applied Biosystems, UK) using standard protocols on the same total RNA samples as the microarray experiment (File S1).

Comparisons between groups were performed using a one-tailed Student's t-test on normalised data, using the comparative Ct method. One of the assays (*Npas4*) was removed from the analysis due to low quality data.

### Ingenuity Pathways Analysis (IPA)

IPA was used to identify functional networks and significantly associated biological pathways amongst the significantly differentially expressed genes (Ingenuity® Systems, www.ingenuity.com) (see File S1 for details). We considered pathways significant at p<0.05.

### Gene Set Enrichment Analysis (GSEA)

To investigate the specific role of food-restriction induced stress response genes in the striatum, and to complement the findings from IPA, we employed GSEA [42]. We used the desktop application of this software for our analysis [43], which is available from the Broad Institute (http://www.broadinstitute.org/gsea/). From the molecular signatures database (MSigDb) (http://www.broadinstitute.org/gsea/msigdb/genesets.jsp) we downloaded the ‘RESPONSE_TO_STRESS’ gene-set, which contains genes annotated by the GO term GO:0006950 and pertains to: “a change in state or activity of a cell or an organism (in terms of movement, secretion, enzyme production, gene expression, etc.) as a result of a stimulus indicating the organism is under stress. The stress is usually, but not necessarily, exogenous (e.g. temperature, humidity, ionizing radiation)”.

For genome-wide gene lists from both Nic vs. Con-Pf and the Con vs. Con-Pf comparisons, we calculated a score that accounted for the strength of both p-value and fold-change (-log10(p-value) x log2(fold-change)). This enabled us to rank the gene lists, whereby we used the GSEA pre-ranked function in the GSEA suite to test for enrichment; using this score we considered the gene-set significant at a p<0.05. Genes that contribute to the enrichment score are defined as those that appear in the ranked list before the maximum enrichment score is reached and therefore describe contributing genes that do not meet the significance thresholds imposed at the single gene level.

### Global DNA methylation analysis by the Luminometric Methylation Assay (LUMA)

Global DNA methylation was quantified using LUMA as previously described [44] (see File S1). Differences in global methylation status were assessed using pair-wise Wilcoxon rank sum tests between groups.

### Assessment of methylation patterns at CpG sites within Fos and Fosb

Changes in gene expression as a result of external stimulation may involve multiple DNA or histone modifications that contribute to an active (or repressive) chromatin state. One such modification is DNA methylation, which is considered a marker of repressed genes.

Promoter CpG sites within *Fos* and *Fosb* were analysed using a bisulphite-based method on the Sequenom mass-array system (see Methods S1 in File S1 and Table S1 in File S1). A Student's t-test at each site for each group-pair was used to assess significant difference between groups.

## Results

### Gestational nicotine treatment and food-restriction affect body weight and solution consumption in dams

Table 1 displays bodyweight, food consumption and solution consumption of dams used in the present study (i.e. a subset of dams described in [17]). As previously described in the behavioural study [17] both the Nic and Con-Pf groups differ significantly for a number of variables compared to controls. Lower dam body weight in both the Nic and Con-Pf groups compared with controls is likely due to a combination of reduced solution and food intake (Table 1). The significantly lower birth weights observed for the Nic group compared with Con (Con = 5.3±0.1 g, Nic = Con = 4.7±0.1 g, p<0.05) in the offspring described in the present study (i.e. experimentally naïve littermates of a subset of dams described in [17]) suggest a specific effect of nicotine on this variable as there was no difference between Con-Pf and Con groups (Con = 5.3±0.1 g, Con-Pf = 5.3±0.1 g, p = NS).

### Microarray analysis reveals limited effects of gestational nicotine treatment but significant effects of gestational food-restriction on striatal mRNA expression profiles

**Figure 1:**
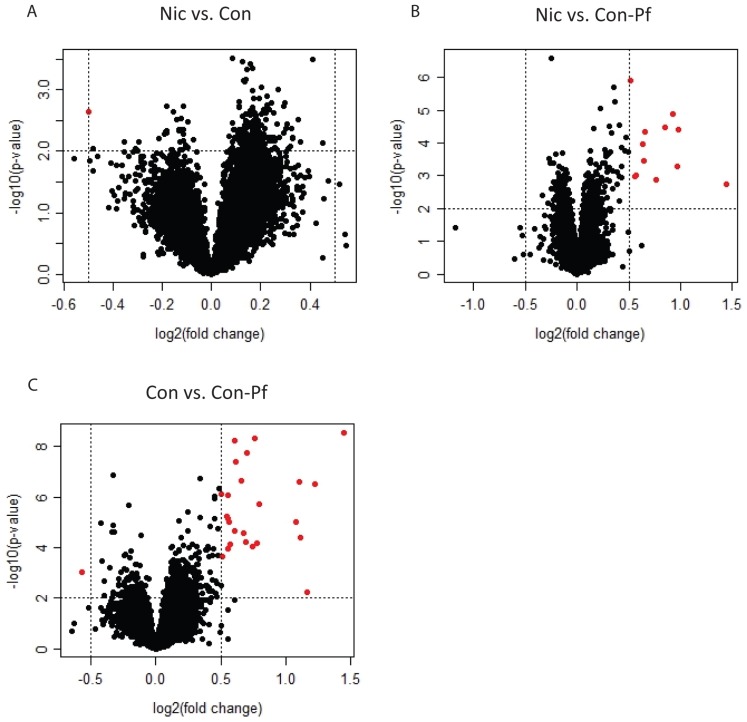
Volcano plots representing group comparisons for all genes included in the analysis. x-axes represent log2 fold-changes and y-axes represent the –log10(p-values) associated with the t-statistic. Vertical dotted lines are positioned at a log2 fold-change of 0.5 or −0.5 and horizontal dotted lines are positioned at the equivalent of p = 0.01. In red are those genes that are differentially expressed at p<0.01 and log2 fold-change>0.5 or <−0.5. A) Nic vs. Con B) Nic vs. Con-Pf and C) Con vs. Con-Pf.

Three group comparisons were performed; Nic versus Con, Con versus Con-Pf and Nic versus Con-Pf (Figure 1). We found little evidence for gene expression differences between the Nic group and the Con group, with just a single gene, *LRRGT00176* reaching significance (log2(fold change) = −0.5, p-value  = 2.20×10^−03^, Table 2). In contrast, we found significant differential expression (p<0.01 and |Fold change ≥0.5|) in both comparisons involving the Con-Pf group (Figure 1). 26 genes were differentially expressed in the Con versus Con-Pf comparison and 12 were differentially expressed in the Nic versus Con-Pf comparison (Table 2) using the designated criteria. These data suggest that imposed food-restriction during gestation is capable of affecting the expression of genes much later in life. To assess whether these changes were true positives, we assessed the significance of these genes using the false discovery rate (FDR) q-value (implemented using the *r q-value* package [45]) as a method for correcting for the number of tests performed (correcting for the original 14,073 probesets analysed) (Table 2). The result of this correction showed low confidence in the finding of differential *LRRGT00176* expression due to gestational nicotine (FDR q = 0.24). However we could be confident that genes called as differentially expressed due to food-restriction using our original criteria were true positives, with multiple genes reaching significance at an FDR q<0.05 (Table 2).

**Table 2:** Results from Student's t-tests for all three group comparisons.

Comparison	Probe-set ID	t-statistic	p-value	FDR q-value	Log2 (FC)	Accession Number	Gene	Description
Nic vs. Con	10868302	3.97	2.20E-03	0.24	−0.50	AY539927	LRRGT00176 mRNA	-
Nic vs. Con-Pf	10727717	4.33	1.79E-03	0.26	1.44	NM_153626	Npas4	neuronal PAS domain protein 4
	10886031	5.80	3.81E-05	0.04	0.98	NM_022197	Fos	FBJ osteosarcoma oncogene
	10899387	5.02	5.26E-04	0.17	0.97	NM_024388	Nr4a1	nuclear receptor subfamily 4, group A, member 1
	10832802	6.39	1.29E-05	0.03	0.92	NM_053633	Egr2	early growth response 2
	10904511	6.61	3.33E-05	0.04	0.85	NM_019361	Arc	activity-regulated cytoskeleton-associated protein
	10868940	3.97	1.32E-03	0.23	0.76	NM_031628	Nr4a3	nuclear receptor subfamily 4, group A, member 3
	10806585	5.68	4.57E-05	0.05	0.65	NM_021836	Junb	jun B proto-oncogene
	10732652	4.62	3.58E-04	0.15	0.64	NM_053769	Dusp1	dual specificity phosphatase 1
	10844339	5.48	1.05E-04	0.09	0.63	NM_145677	Slc25a25	solute carrier family 25
	10940628	4.16	1.00E-03	0.22	0.57	NM_001013146.1	Fosb	Rattus norvegicus FBJ osteosarcoma oncogene B (Fosb)
	10940647	4.18	1.01E-03	0.22	0.56	NM_012954.1	Fosl2	Rattus norvegicus fos-like antigen 2 (Fosl2)
	10834031	7.77	1.25E-06	7.80E-03	0.51	NM_001079893	Dusp14	dual specificity phosphatase 14
Con vs. Con-Pf	10899387	−12.32	2.94E-09	1.49E-05	1.44	NM_024388	Nr4a1	nuclear receptor subfamily 4, group A, member 1/
	10832802	−9.06	3.07E-07	2.27E-04	1.22	NM_053633	Egr2	early growth response 2
	10727717	−3.43	6.01E-03	1.29E-01	1.16	NM_153626	Npas4	neuronal PAS domain protein 4
	10868940	−5.73	3.94E-05	8.09E-03	1.11	NM_031628	Nr4a3	nuclear receptor subfamily 4, group A, member 3
	10904511	−9.25	2.55E-07	2.10E-04	1.10	NM_019361	Arc	activity-regulated cytoskeleton-associated protein
	10886031	−6.68	9.74E-06	2.88E-03	1.07	NM_022197	Fos	FBJ osteosarcoma oncogene
	10806585	−7.30	1.84E-06	8.50E-04	0.79	NM_021836	Junb	jun B proto-oncogene
	10940628	−5.45	6.88E-05	1.30E-02	0.78	NM_001013146.1	Fosb	Rattus norvegicus FBJ osteosarcoma oncogene B (Fosb)
	10760080	−11.43	4.83E-09	1.49E-05	0.76	NM_001002829	Rasl11a	RAS-like family 11 member A
	10940647	−5.35	8.89E-05	1.49E-02	0.74	NM_012954.1	Fosl2	Rattus norvegicus fos-like antigen 2 (Fosl2)
	10872626	−10.37	1.86E-08	3.44E-05	0.70	NM_153727	Gpr3	G protein-coupled receptor 3
	10732652	−5.42	5.86E-05	1.14E-02	0.70	NM_053769	Dusp1	dual specificity phosphatase 1
	10844339	−5.94	2.79E-05	6.08E-03	0.67	NM_145677	Slc25a25	solute carrier family 25
	10834031	−8.78	2.37E-07	2.10E-04	0.66	NM_001079893	Dusp14	dual specificity phosphatase 14
	10716080	−11.05	4.04E-08	5.97E-05	0.62	NM_133578	Dusp5	dual specificity phosphatase 5
	10768332	−11.81	6.03E-09	1.49E-05	0.61	NM_053453	Rgs2	regulator of G-protein signaling 2
	10863676	−6.15	2.26E-05	5.39E-03	0.61	NM_019137	Erg4	early growth response 4
	10815763	−5.36	7.19E-05	1.33E-02	0.57	NM_001107679	Tiparp	TCDD-inducible poly(ADP-ribose) polymerase
	10896793	−6.80	9.41E-06	2.88E-03	0.57	NM_023985	Trib3	tribbles homolog 1 (Drosophila)
	10719432	−5.45	1.10E-04	1.60E-02	0.56	ENSRNOT00000022556	Fosb	FBJ osteosarcoma oncogene B
	10701846	−14.66	6.90E-06	2.32E-03	0.56	NM_053698	Cited2	p300-interacting transactivator, with Glu/Asp-rich carboxy-terminal domain, 2
								
	10800919	−8.48	8.32E-07	4.73E-04	0.55	NM_012551	Egr1	early growth response 1
	10792035	−7.39	5.87E-06	2.28E-03	0.55	NM_022199	Dusp4	dual specificity phosphatase 4
	10780205	−4.99	2.18E-04	2.44E-02	0.51	NM_031056	Mmp14	matrix metallopeptidase 14 (membrane-inserted)
	10710028	−8.00	7.58E-07	4.67E-04	0.51	NM_024362	Arntl	aryl hydrocarbon receptor nuclear translocator-like
	10833811	4.32	9.86E-04	5.84E-02	-0.56	-	-	-

Shown are all genes reaching significance at p<0.01 and |FC≥0.5| ordered by FC. In **bold** are those genes that are up-regulated in both the Nic vs. Con and Con-Pf vs. Con comparisons.

**Figure 2:**
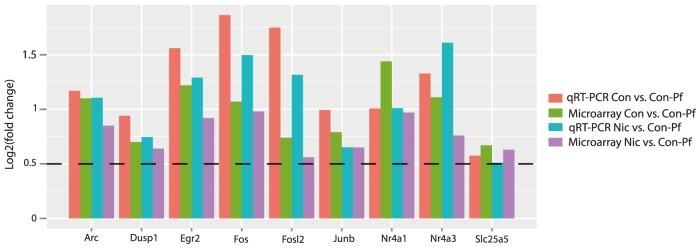
qRT-PCR validation of 9 food-restriction-sensitive genes. The y-axis represents the log2(fold changes) observed in both comparisons involving the food restricted group. Differences in gene expression using qRT-PCR were consistent with microarray data. The dashed line represents the fold change cut-off (log2(fold change) >0.5) used in the microarray analysis.

12 genes were found to be significantly differentially expressed in both the Nic vs. Con-Pf comparison and the Con vs. Con-Pf comparison, suggesting that imposed food-restriction had a dominant effect on striatal gene expression. These genes were predominantly immediate early genes (IEGs) and included; *Fos, Fosl2, Junb, Arc, Egr1, Nr4a1* and *Nr4a3*. To confirm the effects of gestational food-restriction on striatal gene expression we used qRT-PCR to assess differential expression of 9 food-restriction-sensitive genes as a validation set. Significant differential expression in the expected direction was confirmed for all but *Junb* using qRT-PCR (90%, Table 3 and Figure 2).

**Table 3:** qRT-PCR results for differentially expressed genes in microarray analysis.

	Con vs Con-Pf		Nic vs con-Pf	
	T	p.value	t	p.value
Fosl2	−4.46	1.91E-04	3.17	3.10E-03
Fos	−6.10	5.13E-06	4.42	3.42E-04
Nr4a1	−2.96	4.28E-03	3.66	1.11E-03
Nr4a3	−4.46	1.91E-04	3.17	3.10E-03
Slc25a5	−1.72	0.05	1.98	0.04
Arc	−3.78	8.54E-04	3.72	1.11E-03
Junb	−1.78	0.05	1.59	0.07
Dusp1	−3.71	8.40E-04	3.17	2.94E-03
Egr2	−2.39	0.01	1.76	0.05

### Prenatal food-restriction affects genes involved in stress response pathways

**Figure 3:**
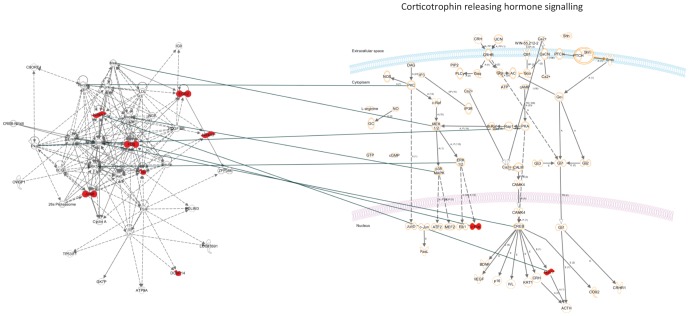
Ingenuity Pathways Analysis of genes identified as differentially expressed in the Nic vs. Con-Pf and the Con vs. Con-Pf comparisons. On the left is the top network identified consisting of 7/12 genes in our list, and on the right is the top associated canonical pathway. Highlighted red are the genes in our list that were over-expressed due to food-restriction.

We aimed to characterise the functional relationship between genes that were regulated by prenatal food-restriction. To this end, we tested the 12 genes that were differentially expressed in both the Nic vs. Con-Pf and Con vs. Con-Pf comparisons for pathway enrichment using Ingenuity pathways analysis (IPA). 7 genes in the set formed an interconnected network (score  = 19, Table 4, Figure 3). Further, functional enrichment analysis revealed that this gene list was enriched for genes involved in the stress response with “Corticotrophin releasing hormone signalling” (ratio  = 2/136, p = 1.98×10^-03^, Figure 3) and “Glucocorticoid receptor signalling” (ratio  = 2/280, p = 8.64×10^-03^) featuring in the top 5 most enriched pathways (Table 5). These enrichment analyses show that while only a few genes are regulated by food-restriction, they appear to be functionally cohesive and related to development of the stress system.

**Table 4:** IPA identified a functional network of genes that included 7 genes differentially expressed due to food-restriction.

Network functions	Molecules in network	Score
Cell Cycle, Gene Expression, Cellular Development	26s Proteasome, Akt, ATP9A, C8ORF4, Creb, CREB-NFkB, CyclinA, DUSP1, DUSP14, EGR2, ERK1/2, FOS, FSH, GK7P, hCG, IG9, Insulin, Jnk, JUNB, LDL, Lh, LOC81691, Mapk, Mek, NFkB (complex), NGF, NR4A1, NR4A3, OVGP1, PDGF BB, PDLIM3, Pka,Pkc(s), TP53I11, ZFP386	19

In **bold** are the genes that were represented from our list of differentially expressed genes.

**Table 5:** The top 5 canonical pathways identified for genes differentially expressed due to food-restriction.

Canonical pathway	p-value	Ratio
Corticotrophin Releasing Hormone Signalling	1.98E-03	2/136 (0.015)
NRF2-mediated Oxidative Stress Response	3.91E-03	2/183 (0.011)
RAR Activation	3.91E-03	2/181 (0.011)
ERK/MAPK Signalling	4.33E-03	2/192 (0.01)
Glucocorticoid Receptor Signalling	8.64E-03	2/280 (0.007)

**Figure 4:**
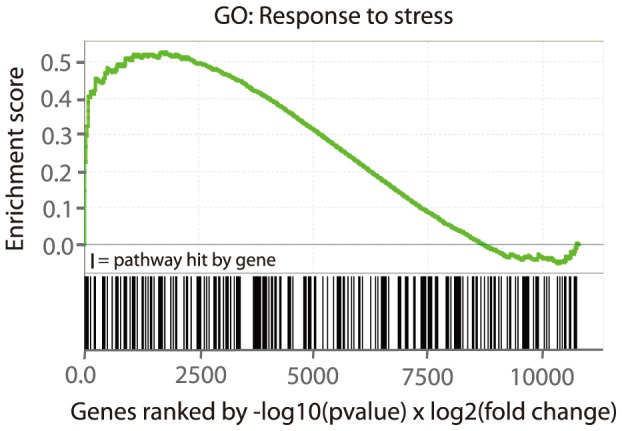
Gene set enrichment analysis (GSEA) of the GO pathway “RESPONSE_TO_STRESS” in the Con vs. Con-Pf comparison. The input gene list was all genes in the microarray analysis ranked by –log10(p-value)×log2(fold change). The enrichment score profile displays an enrichment of pathway hits at the top of the list, suggesting multiple top-ranked genes involved in the “Response to stress” pathway.

Additional evidence for the role of prenatal food-restriction on stress pathways was sought using an independent analysis method. Genome-wide gene lists generated from the Con vs. Con-Pf and Nic vs. Con-Pf comparisons were analysed using Gene Set Enrichment Analysis (GSEA). This method does not rely on user-defined differential expression cut-offs but rather provides a measure of pathway enrichment at the top (or bottom) of a ranked gene list. To specifically test the hypothesis that genes involved in stress responsiveness are affected by gestational food-restriction we used GSEA to assess enrichment for genes in the ‘RESPONSE TO STRESS’ gene ontology (GO) category. We found significant enrichment for this pathway in the Con vs. Con-Pf comparison ranked gene list (p = 0.04, Figure 4) and a trend for significance in the Nic vs. Con-Pf comparison (p = 0.07). The genes that contributed to the enrichment signal in both lists are given in Table 6. As GSEA analysis does not rely on an arbitrary cut-off to be applied to the data it provides additional insight into the potential role of genes that do not reach statistical significance but are, nevertheless, present towards the top of the gene list. Using these analyses we identified further genes that are regulated by food-restriction that were of potential interest. Like the immediate early genes, a subset of these genes is involved in the cellular response to environmental stress. For example we observed up-regulation of *Hspb1*, *Gadd45A/G* and *Ddit3* which are regulated by heat shock, DNA damage and endoplasmic reticulum (ER) stress, respectively [46]-[48]. Further, the anti-proliferation factor *Btg2* is also up-regulated. Interestingly, this gene lies within a quantitative trait locus (QTL) for fear conditioning traits [49], suggesting a link to anxiety-like behaviours. Collectively, these data suggest that gestational food-restriction results in long term up-regulation of multiple stimulation-responsive genes whose role is to induce an appropriate cellular response to a variety of environmental stressors.

**Table 6:** Genes in the core enrichment (i.e. contributing to the maximum enrichment score for the gene set “RESPONSE_TO_STRESS”) from each gene list.

Nic vs. Con-Pf		Con vs. Con-Pf							
Gene	Rank in gene list	Gene	Rank in gene list	Gene	Rank in gene list	Gene	Rank in gene list	Gene	Rank in gene list
FOS	1	FOS	5	CIB1	404	RELA	896	TNFRSF1A	1440
DUSP1	8	DUSP1	15	ATF4	437	SMAD7	899	MT3	1478
GADD45G	32	BTG2	32	TP53I11	452	TGFB1	925	CDO1	1532
BTG2	38	BCL6	45	DNAJB1	463	EEF1E1	1003	AKR1B1	1624
PPP1R15A	50	GADD45A	63	RAD23A	498	PRDX6	1055	STIP1	1638
HSPB1	72	HSPB1	78	DNAJB4	499	ATOX1	1059	UBE2B	1641
PLOD1	78	SCG2	80	DDIT3	615	MSRA	1068	MAP4K2	1662
BCL6	99	PPP1R15A	85	CHRNB2	683	AQP9	1114	XRCC4	1666
PLAT	178	GADD45G	93	TRIAP1	708	GPX3	1166	EIF2B2	1678
SMAD7	204	DNAJB5	134	NDUFS8	709	XRCC6BP1	1275	TP73	1697
SCG2	214	ALKBH1	216	CHRNA4	734	SUMO1	1294		
GADD45A	227	ALOX5AP	240	RTN4RL1	806	NDUFA6	1300		
DNAJB5	302	PLAT	243	PCBP4	871	GAP43	1331		
DDIT3	315	PDLIM1	255	RNF7	895	RPA1	1405		

### Global DNA methylation status and methylation at specific promoters does not explain food-restriction-induced gene expression changes

Given our observations of the up-regulation of stress-response genes due to food-restriction, we hypothesised that early stress leads to epigenetic reprogramming that primes neuronal cells for stressful encounters later in life. To assess the contribution of DNA methylation status on food-restriction-induced gene expression changes we assayed both global DNA methylation changes and locus-specific methylation patterns at *Fos* and *Fosb* genes. We found no evidence for differences in global methylation status between any of the groups analysed (Nic vs. Con-Pf, W = 45, p = 0.70; Con vs. Con-Pf, W = 54, p = 0.50; Nic vs. Con-Pf W = 48, p = 0.28). Further, the majority of CpG sites assessed in the promoter regions of *Fos* and *Fosb* in this study displayed low methylation levels and very little variation in methylation status across samples (Figure S2 in File S1; average variance Fos = 9.6×10^−4^, Fosb = 1.8×10^−3^). We found no evidence for DNA methylation differences between any of the groups for either of the genes tested.

## Discussion and Conclusions

### Prenatal nicotine exposure has limited effects on mRNA expression in the striatum of adolescent rats

We did not find any striking effects of prenatal nicotine exposure on gene expression profiles in the striatum of adolescent rats. However, six previous studies have reported mRNA expression differences due to prenatal exposure to nicotine across various brain regions [18], [20], [24], [25], [50]. We do not replicate the majority of these previously observed nicotine-induced changes (for a summary of previous observations see Table S2 in File S1). The lack of replication does not appear to be due to the thresholds that we used to call genes as differentially expressed as we observe just 12 previously reported genes to be differentially expressed when we apply a more relaxed threshold to the data (p<0.05, no fold change threshold, Table S2 in File S1). This overlap is no greater than we would expect by chance (Nic vs. Con, empirical p = 0.52; Nic vs. Con-Pf, empirical p = 0.69, see Methods S1 in File S1 for statistical analysis). Alternative explanations for the lack of replication are differences in study design and the choice of brain tissue analysed. We can reasonably expect that the effects of prenatal nicotine exposure will vary depending on brain region assayed, as well as developmental time-point. As such it is perhaps not surprising that we fail, in the adolescent striatum, to replicate findings from previous studies. Finally, we cannot exclude potential false negative results in our data that would reflect subtle effects of gestational nicotine on gene expression. However we would require larger sample sizes to robustly identify differentially expressed genes below the significance thresholds that were used in this study.

### Maternal food-restriction has significant effects on mRNA expression in adolescence

In contrast to the effects of prenatal nicotine exposure, we identified striatal gene expression changes following restricted food access to pregnant dams. While it is known that mRNA expression of a selection of genes in both the liver and tissues of the HPA axis is affected by maternal undernutrition [29]–[32], we have provided the first evidence that the striatum is also affected. The set of genes identified contains multiple families of immediate early genes (*Fos, Fosl2, Junb, Arc, Dusp1, Dusp14, Egr2, Nr4a1 and Nr4a3*), which are known to be regulated by multiple external stimuli [51]–[54]. These genes are enriched for two stress-related pathways – “Corticotrophin releasing hormone signalling” and “Glucorticoid receptor signalling” suggestive of food-restriction-induced developmental regulation of the organismal stress system. IEG expression is predominantly activity regulated and levels of *Fos* mRNA are often used as a marker of neuronal activity. As activity-induced transcription factors, IEGs have wide and varied effects on gene regulation that have been associated with multiple brain processes. *Arc* is an important regulator of hippocampal function, where knock-down results in impairment of the maintenance phase of long-term potentiation and hampered long term spatial memory [55]. *Fos* has also been described to have a role in learning and memory, with increased *Fos* expression in the medial prefrontal cortex being linked to aversive learning [56]. Furthermore, administration of amphetamine-based psychostimulants, including cocaine, causes coincident up-regulation of IEG expression and synaptic dopamine release [57]–[62], suggesting an impact of IEG expression on the reward circuitry in the brain. This is of particular interest as these drugs primarily influence neurobehavioural features such as attention, impulsivity and hyperactivity [63]. Given the time from exposure to gene expression measurement and the rapidity of the culling procedure (neck dislocation, 2–3 seconds per animal), we interpret the observed gene expression differences to be due to baseline changes in IEG expression. Nevertheless, we cannot completely exclude the possibility of theoretical pair-feeding-induced increased sensitivity to stress and animals' reactivity to culling. Little is known about the roles of IEGs at baseline, and indeed whether baseline differences in gene expression have an effect on stimulation-induced expression. Adaptation of gene expression to repeated immobilisation stress is observed for *Fos* mRNA in mice, with reduced induction being observed in multiple brain regions upon activation by acute stress [64]. This suggests that *Fos* regulation may be reprogrammed in response to repeated encounters with stressful conditions. Further, It should also be noted that evolutionary adaptations to stressful conditions between yeast species involve baseline differences in the expression of stress-response genes [65]. These adaptive expression profiles may contribute to the differences observed in stimulus-induced expression of such genes [65]. In such a model, stress during early development would prime cells for later stressful encounters through baseline up-regulation of stress-response genes such as *Fos*. Further work is required to describe the effects of gestational food-restriction on IEG-dependent changes in learning, memory, reward and stress responses.

To confirm up-regulation of stress-response genes we performed Gene Set Enrichment Analysis (GSEA). We reasoned that the gene ontology (GO) biological function category “RESPONSE TO STRESS” would be enriched in ranked gene lists from the Con vs. Con-Pf and the Nic vs. Con-Pf comparisons. This was the case for the Con vs. Con-Pf comparison. As this analysis was not restricted to arbitrary differential expression cut-offs, it allowed us to delve further into the data to identify additional potentially important genes that did not meet our threshold requirements. Interestingly, we observed a number of additional genes that appeared near the top of the ranked list and are involved in the cellular response to stress. These genes include *Hspb1*, *Gadd45A/G* and *Ddit3* that are responsive to heat shock, DNA damage/growth arrest and endoplasmic reticulum (ER) stress [46]–[48]. The reason for their persistent up-regulation is not entirely clear. However, we speculate that it may be similar to the increased baseline expression of IEGs – early developmental induction of the stress response through food-restriction induces reprogramming and an altered baseline level of mRNA expression. Food-restriction-induced early induction of stress response genes is likely to have consequences on normal brain development. For example, over-expression of *Gadd45a* in the developing mouse cortex is associated with decreased neurite complexity, soma hypertrophy and increases in cell death [66]. Chronic up-regulation during adolescence may have further detrimental consequences related to neuronal function and cell death. *Ddit3* is responsive to ER stress, whereby exacerbation of ER stress using 1-Methyl-4-phenylpyridinium ion (MPP+) increases its expression level [67] and may contribute to Parkinson's disease. Again, we do not know how baseline up-regulation of *Ddit3* affects the adolescent brain, although we speculate that defective ER stress signalling pathways will affect neuron integrity. Interestingly, changes in genes involved in the cellular response to stress may also have an effect on the organismal response to stress. This is evident from a recent study describing the role of mutations in *C. elegans* DNA repair and apoptotic pathway genes conferring resistance to environmental stressors such as heat shock and osmotic stress [68]. Early developmental alterations in similar pathways may display similar effects. Future work should aim to further understand whether food-restriction during gestation is capable of altering both cellular and organismal responses to stress stimuli.

Recent epigenetic studies have provided a framework through which the environment can shape gene expression patterns in later life and alter offspring behaviour [69]–[71]. For example, maternal protein restriction is associated with a reduction in DNA methylation at the promoter of the Cyclin-dependent kinase inhibitor 1C (*Cdkn1c*) promoter [72] which results in an increase in mRNA levels. This suggests that early protein deprivation is associated with epigenetic reprogramming of a certain set of genes whose dysregulation may be responsible for alterations in behaviour. We hypothesised that food-restriction-induced up-regulation of IEG expression may be due to early acquired epigenetic marks that persist into adolescence and explain the observed differences in mRNA regulation. However, we failed to find any *global* DNA methylation effects or changes in patterns of DNA methylation across two regions within *Fos* or *Fosb*. Our results on global DNA methylation are consistent with studies in humans [73] and suggest that specific regulatory regions govern the observed gene expression patterns. Given the very low and invariant methylation levels at *Fos* and *Fosb* promoters it is was unlikely that they would harbor critical sites of regulation. Nevertheless, these data do not rule out the potential of DNA methylation or alternative epigenetic marks to set up an early program of gene expression that persists into adolescence.

### Limitations

The unexpected limitation of the current study was a potential interaction between prenatal undernutrition and different stress levels induced by either nicotine exposure or enforced food availability. Our intention was to control for reduced food intake experienced in the Nic group by using a pair-feeding protocol. However, as gene expression changes in Con-Pf group were seen over and above any small nicotine-induced differences it may suggest either ameliorating effects of nicotine or existence of additional factors, e.g., higher stress, in pair-fed animals. Thus, we were able to control for undernutrition (using Con-Pf) as well as the stress induced by pair-feeding (using Con), but not for a potential stress x undernutrition interaction. Unfortunately, there is no obvious way to do this in one experiment. Nevertheless, under our study conditions, there were no strong effects of gestational nicotine exposure on striatal gene expression.

### Conclusion

Our work has highlighted a role for maternal food-restriction on the long-term regulation of immediate early genes and stress-response genes in the striatum of adolescent rats. Future studies should therefore focus on establishing the link between food-restriction, stress, IEG expression and phenotypic outcome.

### Data availability

Microarray data are available from the Gene Expression Omnibus (GEO) (http://www.ncbi.nlm.nih.gov/geo/) under accession number GSE50607.

## Supporting Information

File S1 **Supporting information.**
(DOCX)
