# Supplementary material for: Long-Term Effects of Gestational Nicotine Exposure and Food-Restriction on Gene Expression in the Striatum of Adolescent Rats
Source: PLoS One. 2014 Feb 19;9(2):e88896. doi: 10.1371/journal.pone.0088896 (PMC3929494; doi:10.1371/journal.pone.0088896)
Supplement: File S1 — Supporting information. (DOCX) [file pone.0088896.s001.docx]

**Supporting Information File S1**

**Methods S1**

***Sample preparation***

Purity of total RNA was assessed via the 260/280-wavelength ratio using a NanoDrop spectrophotometer. All ratios were of acceptable quality (range 2.04 – 2.19). The integrity of total RNA was assessed using the Agilent RNA 6000 pico kit run on a 2100 bioanalyser according to the manufacturer’s guidelines (Agilent, UK). RNA integrity numbers (RIN) were generated using Agilent 2100 Expert software. RIN’s for the sample suggested good quality RNA with one sample being below an adequate quality (RIN = 1.4). With this sample removed, RINs ranged from 8.8 – 9.8. Given the overall good quality of the data, a low RIN for one sample was likely due to technical issues associated with chip loading and this sample was kept in for downstream processing.

***Microarray Processing***

100ng of starting total RNA was used to produce second cycle cDNA for fragmentation and labeling using the Ambion WT expression kit following the manufacturer’s guidelines (Ambion, UK). Fragmentation and labeling of second cycle cDNA was performed using the Affymetrix GeneChip WT terminal labeling and hybridization kit (Affymetrix, UK). The Affymetrix GeneChip wash and stain kit (Affymetrix, UK) was used to hybridize samples to Affymetrix Rat GeneChip 1.0ST arrays. Washing and staining of the arrays was carried out on Affymetrix fluidics modules following Affymetrix protocols. Following array scanning, Affymetrix GeneChip Operating Software (GCOS) was used to visualize the array images and provide an initial check of data quality (smudges on the chip etc). Two samples at this stage were removed from further analysis because of poor data quality.

Quality control checks at each stage of processing were employed to ensure good quality throughout the procedure i.e. cRNA and 2^nd^ cycle cDNA yields and quality were checked using a NanoDrop spectrophotometer; and the size distribution of cRNA, 2^nd^ cycle cDNA and fragmented cDNA were assessed using the Agilent 2100 bioanalyser.

***Quantitative reverse transcription PCR (qRT-PCR) analysis of differentially expressed genes***

Genes chosen for the validation were Fos, Fosl2, Arc, Nr4a1, Nr4a3, Slc25a25, Junb, Dusp1 and Egr2. 3ug total RNA was first-strand reverse transcribed in 20ul reactions using oligodT priming and Moloney Murine Leukaemia Virus (MMLV) (qScript reverse transcription kit, PrimerDesign, UK). In order to identify the most stable housekeeping genes for normalisation, we performed GeNorm analysis on 5 samples from each group, using 10 commonly used HK genes.

Each 20ul assay was run in duplicate using the ABI7900HT system. Mean threshold cycle (Ct) values across duplicates were taken and relative quantities calculated. GeNorm, a Visual Basic application tool in excel was used to statistically model the stability of the HK genes for accurate normalisation of target genes as described elsewhere ([1](#_ENREF_1)). The two most stable HK genes were Atp5b and Cyc1. We chose to use Atp5b for normalisation of our target genes.

Target gene assays were supplied by Applied Biosystems, UK, and each assay was run in triplicate in 20ul reactions with the following cycling conditions; 1 cycle of 95 ^o^C for 10 mins, and 40 cycles of 95 ^o^C for 15 secs, 60 ^o^C for 1 min, using the ABI7900HT system.

***Statistical analysis of qRT-PCR data***

The grubb’s method was used to identify outliers from triplicate samples ([2](#_ENREF_2)), which were removed from further analysis. For the remaining samples, arithmetic means were taken across replicates and the comparative Ct method (ΔΔCt ) was used to normalize expression data of target genes to Atp5b for each sample. Relative quantities for each sample were calculated using the equation 2^ΔCt^. These values were normalized to Atp5b expression (normalization factor) to provide normalized relative quantities (NRQ).

Data were tested for normality using the Shapiro-Wilk test. Normalised data for Fosl2, Fos, Nr4a3, Arc, Dusp1 and Egr2 were rejected for normality at the p<0.05 level and data were log10 transformed for a more normal distribution. Data for the remaining genes; Nr4a1, Slc25a25 and Junb were analyzed using raw normalized scores.

***Ingenuity Pathways Analysis (IPA)***

A data set containing HUGO gene symbols was uploaded into the application. Genes significant at p<0.01 with a log2 fold change of >0.5 (or <-0.5) were input into the analysis. These analysis methods allow for the identification of interconnectedness of the molecules of interest (networks), as well as any biological pathways that are enriched amongst these molecules (canonical pathways analysis). Networks are generated *de novo* using the dataset that is provided by the user. Networks are then algorithmically generated, maximizing the interconnectedness between network eligible molecules relative to their connectedness with all other molecules in the knowledge base for information on the network generating algorithm see (https://analysis.ingenuity.com/pa/info/help/help.htm#ipa_help.htm). In contrast to network analysis, canonical pathways are generated *prior* to data input based on the literature and KEGG legend. In this way, input molecules are tested for enrichment in predefined metabolic and signaling pathways. P-values are then generated for each canonical pathway using a right-tailed Fisher’s exact test (see https://analysis.ingenuity.com/pa/info/help/help.htm#ipa_help.htm). We considered pathways significant at p<0.05.

***Gene set enrichment analysis (GSEA)***

This method takes advantage of the complete probability distribution from the analyses, and therefore is not restricted by a user-defined significance threshold. GSEA assesses the significance of individual, predefined gene sets in a gene list ranked by any particular statistic, which is described in detail elsewhere. A weighted Kolmogorov-Smirnov test is used to determine whether the input distribution is significantly different to the null distribution (based on 1000 permutations of genes) ([3](#_ENREF_3)).

***Global DNA methylation analysis by the Luminometric Methylaytion Assay (LUMA)***

A total of 300ng genomic DNA was cleaved with HpaII/EcoRI or MspI/EcoRI in separate reactions, in duplicate. After the digestion step, the amount of cleavage was quantified by pyrosequencing. DNA methylation was defined from the HpaII/MspI ratio: fully methylated DNA gives a ratio that approaches 0 whereas if methylation is completely absent the ratio approaches 1.

***Assessment of methylation patterns at CpG islands within Fos and Fosb***

500ng of Genomic DNA was treated with sodium bisulfite using the EZ-96 DNA Methylation Kit (Zymo Research, CA, USA) following the manufacturer’s guidelines. Bisulfite-PCR amplification of the regions of interest (See Supplementary material, Table S1 for primer and target sequences) were carried out in duplicate for each sample using cycling conditions of 47 cycles with an annealing temperature of 56°C. Quantitative DNA methylation analysis was conducted on pooled duplicate PCR products using the Sequenom EpiTYPER sys­tem (Sequenom Inc., CA, USA), which utilises base-specific cleavage fol­lowed by MALDI-TOF mass spectrometry, and is described in detail elsewhere ([4](#_ENREF_4)). Positive controls, including both artificially methylated and artificially unmethylated samples were included in all experi­mental procedures to ensure accuracy in the quantification. Multiple CpG sites were analysed for each assay, and to ensure good data quality, sites were removed from the analysis if they showed >75% missing data, and individual samples were removed if they had >75% CpG sites missing. The total number of CpG sites analysed were 16 for Fos and 11 for Fosb. Differences in methylation status between groups were assessed using a pair-wise Student’s t-test.

**Table S1.** Primers used for the amplification of bisulphite treated DNA for two differentially expressed genes identified through microarray analysis

| *Gene* | *Left Primer* | *Right Primer* | Target Sequence |
| --- | --- | --- | --- |
| Fos | GGAGATTTTTTAAGATTTTAATTGTGA | ACAAAAAAACCCATACTAAAAAAAA | GGAGACCTTCTAAGATCCCAATTGTGAACACTCATAGGTGAAAGTTACAGACTGAGAC  GGGGGTTGAGAGCCTGGGGCGTAGAGTTGATGACAGGGAGCCCGCAGAGGGCATTCG  GGAGCGCTTTCCCCCCTCCAGTTTCTCTGTTCCGCTCATGACGTAGTAAGCCATTCAAGCG  CTTCTATAAAGCGGCCAGCTGAGGCGCCTACTACTCCAACCGCGATTGCAGCTAGCAACTG  AGAAGACTGGATAGAGCCGGCGGAGCCGCGAACGAGCAGTGACCGCGCTCCCACCCAGCTCTGCTCTGCAGCTCCCACCAGTGTCTACCCCTGGACCCCTCGCCGAGCTTTGCCCAAACCACGACCATGATGTTCTCGGGTTTCAACGCGGACTACGAGGCGTCATCCTCCCGCTGCAGTAGCGCCTCCCCGGCCGGGGACAGCCTTTCCTACTACCATTCCCCAGCCGACTCCTTCTCCAGCATGGGCTCCCCTGT |
| Fosb | GGGAAGAATTATAAAAAGTAAGGAAAGG | ACAAAAAAAATCCCAAAAAAACTTC | GGGAAGAACCACAAAAAGCAAGGAAAGGAGCTACGCTCTACAAATAGGAAGGGAACGTT  GATTGGCTAAAGGCAGCTCCGCGACAGCCAATCACAAAGCCGCGCTGCCCCCTCCCTTAGCAACGTGGCCTCGGCGTTCCAAAATAGAACGTGCCCGGGACGTCAGGGGCGGGGCGGGCGGAGGGGGCGCCGCGCGCCGTGCGTGCCCCGCGTCAGACGGAGGATTCCAGCGCGTCAGCCTTTACGCACGGGTCCCCCGTTACGTCTCCGCGCAGGAGGCGGAGGCAGCGTGACCGGGGTTCGAGGCCCGAACTATTGCAGGGTGGAGGGTGACCGCGGACCTGCAGTCCGCGCTTGGAAGGAGGAGGGAAGAAGAGGAGGCTACTGGAAGCTTCTCTGGGATTTTTCCTGT |

***Calculating the probability of overlap with candidate genes from the literature***

In order to see whether there was any signal in genes that have previously been reported to be differentially expressed due to gestational nicotine, we identified candidate genes based on positive reports from the literature (Table S2). We accepted genes that had been shown to be differentially expressed in any brain region, male or female, any nicotine administration protocol, any mRNA detection technique and any postnatal age. Studies were identified using the search terms ‘gestational nicotine gene expression’ and manually filtered to fit the above criteria.

We used permutation testing (implemented in R2.13.0) to identify the probability of observing our intersections with the candidate gene list. Specifically, we took a fixed set of 35 genes and for each comparison involving the Nic group, we identified the number of genes that were significantly differentially expressed at p<0.05 i.e. marginal evidence for differential expression. We then randomly sampled this number of genes (n = 1058 for Nic vs. Con and n = 965 for Nic vs. Con-Pf) from the total list and calculated the number of genes that were present in the candidate list. After 10,000 permutations we derived a p-value based on this null distribution.

**RESULTS S1**

***Quality Control of Expression Data***

A box plot of the relative log intensities of probe-set summaries was used to identify any clear outliers. One sample was an obvious outlier and was removed from the study. The remaining 25 arrays had comparable relative signal intensities (Figure S1). The positive versus negative control area under the curve ratios showed good data quality, with values ranging from 0.91 to 0.93. Signal intensities for bacterial spikes (*BioB*, *BioC*, *BioD* and *Cre*) also displayed a characteristic profile of intensity in the order: *BioB* < *BioC* < *BioD* < *Cre*. The signal intensities of each spike probe for each array showed good inter-array consistency and accurate intra-array spike order. Probe-set summaries were used for all remaining downstream analyses.

Figure S1


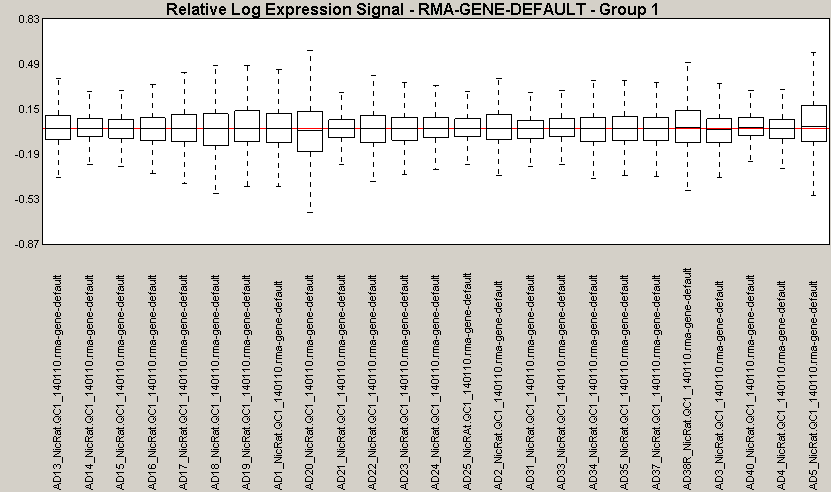


**Figure S1:** Relative Log Expression signals for 25 arrays. Each box represents an individual array and the y-axis is the relative log expression value (output from Affymetrix Expression console).

***Assessment of methylation patterns at CpG islands within Fos and Fosb***

Figure S2


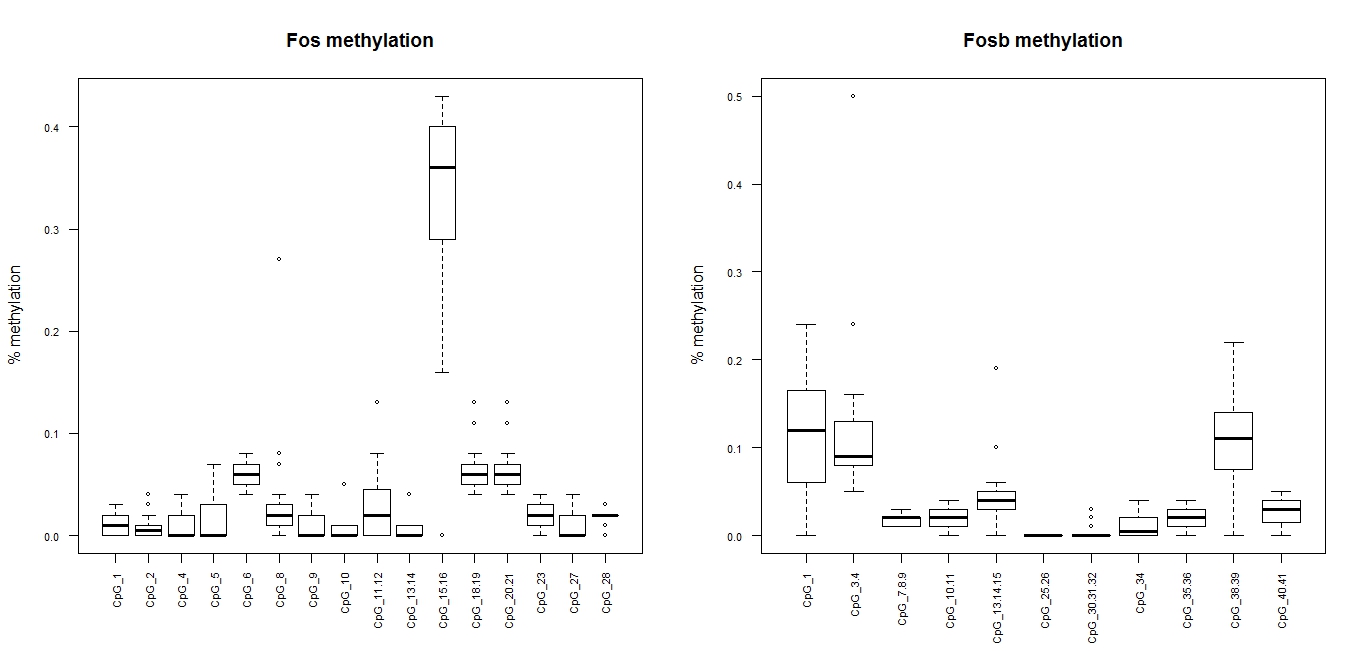


**Figure S2.** Boxplots representing the percentage methylation at each analysed CpG site for two differentially expressed genes. X-axis is CpG site and y-axis displays the percentage methylation.

**Table S2:** Genes previously found to be differentially expressed due to prenatal nicotine exposure and overlap with the results from our study. Genes in **bold** are significant at p<0.05. Basic study designs are also presented. Fc = fold change; VTA = Ventral tegmental area; Nac = nucleus accumbens; Pfc = prefrontal cortex; Amy = Amygdala; Cpu = Caudate Putamen; Il = infralimbic cortex. Na = gene was not represented in the analysed data set (not on array or filtered out in preprocessing).

| **Results from previous studies** | | | | | | | | **Results from this study (Striatum)** | | |
| --- | --- | --- | --- | --- | --- | --- | --- | --- | --- | --- |
| *Reference* | *Nicotine administration*  *Method* | *Controls*  *Used* | *Cross-fostered* | *Sex* | *Age* | *DEG* | *Brain region* | *Nic vs. Con* | *Nic vs. Con-pf* | *Con vs. Con-pf* |
| ([5](#_ENREF_5)) | Osmotic minipump  (2 and 6mg/kg/day)  G4-G12 and G21 | Saline vehicle | No | M/F | P2 | Fos | BS(6), FB(2/6) | Fc = 0.1 p = 0.46 | **Fc = 0.99 p = 3.81E-05** | **Fc = 1.07 p = 9.74E-06** |
|  |  |  |  |  |  |  |  |  |  |  |
| ([6](#_ENREF_6)) | Osmotic minipump  (2mg/kg body weight/day) G4-22, PN2-14 | Saline vehicle pair-fed to nicotine group | No | M/F | PN35 | Chrna3 | VTA, Nacc Core | **Fc = 0.20 p = 0.04** | Fc = -0.15 p = 0.10 | Fc = 0.05 p = 0.37 |
|  |  |  |  |  |  | Chrna4 | VTA | Fc = 0.28 p = 0.07 | Fc = -0.14 p = 0.30 | **Fc = 0.15 p = 0.02** |
|  |  |  |  |  |  | Chrna5 | VTA | Na | Na | Na |
|  |  |  |  |  |  | Chrnb4 | VTA | Fc = 0.05 p = 0.14 | Fc = -0.0000, p = 0.96 | Fc = 0.05 p = 0.22 |
|  |  |  |  |  |  |  |  |  |  |  |
| ([7](#_ENREF_7)) | Osmotic minipump (3mg/kg/day)  G4-G18 | Saline vehicle | Yes | M | P38-42 | Fos | Ilc, Nac | Fc = 0.1 p = 0.46 | **Fc = 0.99 p = 3.81E-05** | **Fc = 1.07 p = 9.74E-06** |
|  |  |  |  |  |  |  |  |  |  |  |
| ([8](#_ENREF_8)) | Osmotic minipump (3mg/kg.d) G4-18 | Saline vehicle | Yes | F | PN35 | Nrxn3 | PFc | Fc = -0.07 p = 0.09 | Fc = -.02 p = 0.50 | **Fc = -0.10 p = 0.05** |
|  |  |  |  |  |  |  |  |  |  |  |
|  |  |  |  |  |  | Nlgn1 | Cpu | Fc = -0.12 p = 0.17 | Fc = -0.06 p = 0.16 | Fc = -0.18 p = 0.06 |
|  |  |  |  |  |  | Ncam1 | Amy | Fc = -0.04 p = 0.43 | Fc = 0.01 p = 0.66 | Fc = -0.03 p = 0.54 |
|  |  |  |  |  |  | Cntn4 | Cpu, PFc | Fc = -0.02 p = 0.79 | Fc = -0.01 p = 0.80 | Fc = -0.03 p = 0.64 |
|  |  |  |  |  |  | Cntn5 | Cpu | Fc = 0.02 p = 0.69 | Fc = -0.03 p = 0.60 | Fc = -0.01 p = 0.88 |
|  |  |  |  |  |  | Cntn6 | Cpu | Fc = 0.01 p = 0.84 | Fc = 0.06 p = 0.30 | Fc = 0.07 p = 0.34 |
|  |  |  |  |  |  | Dscam | Cpu, PFc | Fc = -0.03 p = 0.68 | Fc = -0.012 p = 0.73 | Fc = -0.05 p = 0.47 |
|  |  |  |  |  |  | Pecam | Amy | Na | Na | Na |
|  |  |  |  |  |  | Postn | Cpu, Nac, PFc | Na | Na | Na |
|  |  |  |  |  |  | Actn1 | Cpu | Fc = -0.05 p = 0.18 | Fc = 0.005 p = 0.83 | Fc = -0.05 p = 0.22 |
|  |  |  |  |  |  | Cdh13 | Cpu | Fc = -0.05 p = 0.37 | Fc = -0.02 p = 0.64 | Fc = -0.07 p = 0.18 |
|  |  |  |  |  |  | Ctnna1 | Cpu | **Fc = -0.12 p = 0.02** | Fc = 0.06 p = 0.11 | Fc = -0.06 p = 0.19 |
|  |  |  |  |  |  | Ctnna2 | Cpu | Fc = -0.05 p = 0.47 | **Fc = -0.08 p = 0.04** | Fc = -0.13 p = 0.08 |
|  |  |  |  |  |  | Ctnnb1 | Cpu | Fc = -0.07 p = 0.37 | Fc = -0.02 p = 0.58 | Fc = -0.09 p = 0.20 |
|  |  |  |  |  |  | Ctnnd2 | Cpu | Fc = 0.01 p = 0.74 | Fc = -0.03 p = 0.06 | Fc = -0.02 p = 0.37 |
|  |  |  |  |  |  | Bai3 | Cpu | Fc = -0.04 p = 0.20 | Fc = -0.04 p = 0.23 | **Fc = -0.08 p = 0.04** |
|  |  |  |  |  |  | Lphn3 | Cpu, Amy | Fc = -0.14 p = 0.09 | Fc = 0.001 p = 0.97 | Fc = -0.14 p = 0.10 |
|  |  |  |  |  |  | Ptprd | Nac | Fc = -0.09 p = 0.07 | Fc = 0.03 p = 0.26 | Fc = -0.06 p = 0.23 |
|  |  |  |  |  |  | Csmd1 | Cpu | Fc = -0.17 p = 0.14 | Fc = -0.03 p = 0.45 | Fc = -0.20 p = 0.10 |
|  |  |  |  |  |  | Sgcz | Amy | Fc = 0.07 p = 0.34 | Fc = 0.11 p = 0.19 | **Fc = 0.19 p = 0.05** |
| ([9](#_ENREF_9)) | Osmotic minipump (3mg/kg.d)  G4-18 | Saline vehicle | Yes | F | PN35 | BdnF | Nac, PVN, Striatum | Na | Na | Na |
|  |  |  |  |  |  | IgF1r | Nac, PVN | Fc = -0.17 p = 0.09 | Fc = -0.01 p = 0.75 | Fc = -0.19 p = 0.07 |
|  |  |  |  |  |  | Ins | Nac, Striatum | Na | Na | Na |
|  |  |  |  |  |  | Itgb5 | Nac, PVn, Striatum | Na | Na | Na |
|  |  |  |  |  |  | PdgFa | Amy, PVN | Fc = 0.03 p = 0.46 | Fc = -0.02 p = 0.46 | Fc = 0.01 p = 0.8 |
|  |  |  |  |  |  | VEGFA | PVN | Fc = -0.08 p = 0.15 | Fc = 0.02 p = 0.76 | Fc = -0.07 p = 0.08 |
|  |  |  |  |  |  | Map2k6 | PVN | Fc = 0.0001 p = 1.00 | Fc = -0.04 p = 0.50 | Fc = -0.04 p = 0.46 |
|  |  |  |  |  |  | Mapk8 | Nac | Fc = -0.07 p = 0.35 | Fc = -0.04 p = 0.13 | Fc = -0.10 p = 0.16 |
|  |  |  |  |  |  | Prkca | Striatum | Fc = -0.04 p = 0.44 | Fc = -0.07 p = 0.02 | Fc = -0.11 p = 0.08 |
|  |  |  |  |  |  | Prkce | Nac, PFc | Fc = -0.02 p = 0.54 | Fc = -0.01 p = 0.72 | Fc = -0.04 p = 0.37 |
|  |  |  |  |  |  | Fos | PVN | Fc = 0.1 p = 0.46 | **Fc = 0.99 p = 3.81E-05** | **Fc = 1.07 p = 9.74E-06** |
|  |  |  |  |  |  | Mef2c | Amy, Nac, Striatum | **Fc = 0.14 p = 6.09E-03** | Fc = -0.06 p = 0.19 | Fc = 0.09 p = 0.10 |
|  |  |  |  |  |  | Tp53 | Nac | Fc = 0.12 p = 0.12 | Fc = -0.10 p = 0.11 | Fc = 0.02 p = 0.74 |
|  |  |  |  |  |  | TnF | Striatum | Na | Na | Na |
|  |  |  |  |  |  | Tnfrsf1a | Striatum | **Fc = 0.19 p = 7.31E-03** | Fc = -0.08 p = 0.15 | **Fc = 0.11 p = 0.05** |
|  |  |  |  |  |  | Tnfsf6 | Amy, PFc | Na | Na | Na |
|  |  |  |  |  |  | Casp2 | PFc, Striatum | Fc = 0.03 p = 0.61 | Fc = -0.03 p = 0.53 | Fc = -0.0008 p = 0.99 |
|  |  |  |  |  |  | Casp3 | PFc, Striatum | Fc = 0.11 p = 0.08 | Fc = -0.07 p = 0.26 | Fc = 0.04 p = 0.59 |
|  |  |  |  |  |  | Casp8 | Striatum | Na | Na | Na |
|  |  |  |  |  |  |  |  |  |  |  |
| ([10](#_ENREF_10)) | Drinking water  (0.04ml/kg body weight/day) | Drinking water | Yes | M | PN140 | Drd5 | Striatum | Fc = -0.15 p = 0.18 | Fc = -0.05 p = 0.58 | Fc = -0.2 p = 0.08 |
|  |  |  |  |  |  |  |  |  |  |  |
|  |  |  |  |  |  |  |  |  |  |  |

References

1. Vandesompele J, De Preter K, Pattyn F, Poppe B, Van Roy N, De Paepe A, et al. (2002): Accurate normalization of real-time quantitative RT-PCR data by geometric averaging of multiple internal control genes. *Genome Biol*. 3:RESEARCH0034.

2. Burns MJ, Nixon GJ, Foy CA, Harris N (2005): Standardisation of data from real-time quantitative PCR methods - evaluation of outliers and comparison of calibration curves. *BMC Biotechnol*. 5:31.

3. Subramanian A, Tamayo P, Mootha VK, Mukherjee S, Ebert BL, Gillette MA, et al. (2005): Gene set enrichment analysis: a knowledge-based approach for interpreting genome-wide expression profiles. *Proc Natl Acad Sci U S A*. 102:15545-15550.

4. Coolen MW, Statham AL, Gardiner-Garden M, Clark SJ (2007): Genomic profiling of CpG methylation and allelic specificity using quantitative high-throughput mass spectrometry: critical evaluation and improvements. *Nucleic Acids Res*. 35:e119.

5. Slotkin TA, McCook EC, Seidler FJ (1997): Cryptic brain cell injury caused by fetal nicotine exposure is associated with persistent elevations of c-fos protooncogene expression. *Brain Res*. 750:180-188.

6. Chen H, Parker SL, Matta SG, Sharp BM (2005): Gestational nicotine exposure reduces nicotinic cholinergic receptor (nAChR) expression in dopaminergic brain regions of adolescent rats. *Eur J Neurosci*. 22:380-388.

7. Park MK, Loughlin SE, Leslie FM (2006): Gestational nicotine-induced changes in adolescent neuronal activity. *Brain Res*. 1094:119-126.

8. Cao J, Dwyer JB, Mangold JE, Wang J, Wei J, Leslie FM, et al. (2011): Modulation of cell adhesion systems by prenatal nicotine exposure in limbic brain regions of adolescent female rats. *Int J Neuropsychopharmacol*. 14:157-174.

9. Wei J, Wang J, Dwyer JB, Mangold J, Cao J, Leslie FM, et al. (2011): Gestational nicotine treatment modulates cell death/survival-related pathways in the brains of adolescent female rats. *Int J Neuropsychopharmacol*. 14:91-106.

10. Schneider T, Ilott N, Brolese G, Bizarro L, Asherson PJ, Stolerman IP (2011): Prenatal exposure to nicotine impairs performance of the 5-choice serial reaction time task in adult rats. *Neuropsychopharmacology*. 36:1114-1125.
